# Enzymes of an alternative pathway of glucose metabolism in obligate methanotrophs

**DOI:** 10.1038/s41598-021-88202-x

**Published:** 2021-04-22

**Authors:** Olga N. Rozova, Galina A. Ekimova, Nikolai V. Molochkov, Alexander S. Reshetnikov, Valentina N. Khmelenina, Ildar I. Mustakhimov

**Affiliations:** 1 Federal Research Center “Pushchino Scientific Center for Biological Research of the Russian Academy of Sciences”, G.K. Skryabin Institute of Biochemistry and Physiology of Microorganisms, Russian Academy of Sciences, Pushchino, Moscow Region Russia 142290; 2 Institute of Theoretical and Experimental Biophysics, Russian Academy of Sciences, Pushchino, Moscow Region Russia 142290

**Keywords:** Microbiology, Bacterial physiology, Biochemistry, Enzymes

## Abstract

Aerobic methanotrophic bacteria utilize methane as a growth substrate but are unable to grow on any sugars. In this study we have shown that two obligate methanotrophs, *Methylotuvimicrobium alcaliphilum* 20Z and *Methylobacter luteus* IMV-B-3098, possess functional glucose dehydrogenase (GDH) and gluconate kinase (GntK). The recombinant GDHs from both methanotrophs were homotetrameric and strongly specific for glucose preferring NAD^+^ over NADP^+^. GDH from *Mtm. alcaliphilum* was most active at pH 10 (*V*_max_ = 95 U/mg protein) and demonstrated very high *K*_m_ for glucose (91.8 ± 3.8 mM). GDH from *Mb. luteus* was most active at pH 8.5 (*V*_max_ = 43 U/mg protein) and had lower *K*_m_ for glucose (16 ± 0.6 mM). The cells of two *Mtm. alcaliphilum* double mutants with deletions either of the genes encoding GDH and glucokinase (*gdh*^─^/*glk*^─^) or of the genes encoding gluconate kinase and glucokinase (*gntk*^─^/*glk*^─^) had the lower glycogen level and the higher contents of intracellular glucose and trehalose compared to the wild type strain. The *gntk*^─^/*glk*^─^ knockout mutant additionally accumulated gluconic acid. These data, along with bioinformatics analysis, demonstrate that glycogen derived free glucose can enter the Entner–Doudoroff pathway or the pentose phosphate cycle in methanotrophs, bypassing glycolysis via the gluconate shunt.

## Introduction

**Figure 1:**
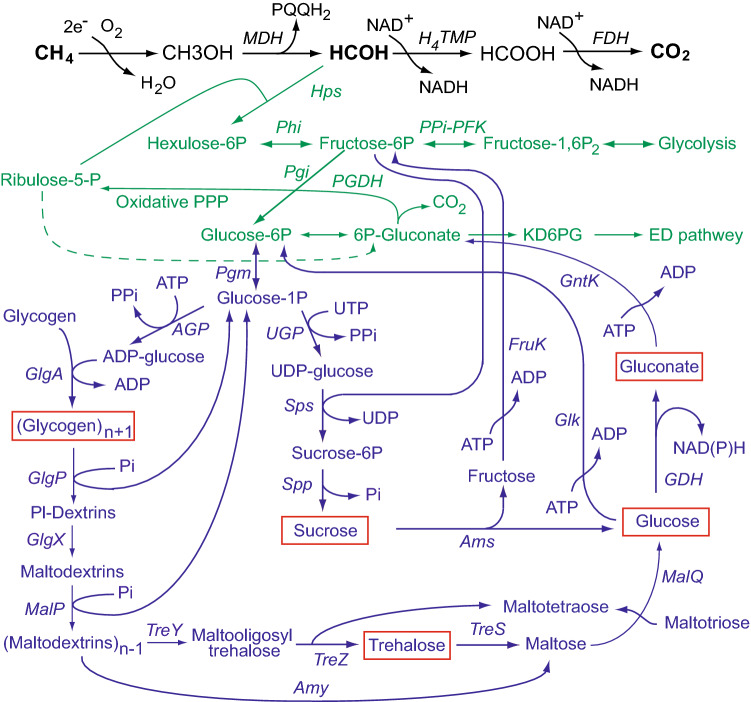
Schematic pathways for glycogen synthesis and degradation in *Mtm. alcaliphilum* 20Z. The methane oxidation reactions are black colored, the carbon assimilation pathways are green, the carbohydrate metabolism reactions are blue. *MMO,* methane monooxygenase; *MDH,* methanol dehydrogenase; *FDH,* formate dehydrogenase; *HPS,* hexulose-phosphate synthase; *HPI,* hexulosephosphate isomerase; *PGI,* phosphoglucose isomerase; *PGM,* phosphoglucose mutase; *Glk,* glucokinase; *GntK,* gluconate kinase; *FruK,* fructokinase; *UGP,* uridine glucopyrophosphorylase; *AGP,* adenosine glucopyrophosphorylase; *GlgA,* glycogen synthase; *GlgP,* glycogen phosphorylase; *GlgX,* debranching enzyme; *TreY,* maltooligosyl trehalose synthase; *TreZ,* trehalohydrolase; *MalP,* maltodextrin phosphorylases; *TreS,* trehalose synthase; *MalQ,* alpha-glucanotransferase; *Ams,* amylosucrase; *Sps,* sucrose-phosphate synthase; *Spp,* sucrose-phosphate phosphatase; *GDH,* glucose dehydrogenase; *Gnl,* lactonase. The genome analysis was carried out using NCBI and MicroScope databases (http://www.ncbi.nlm.nih.gov, https://mage.genoscope.cns.fr).

Aerobic methanotrophs are a highly specialized group of bacteria that utilize methane as an energy and carbon source. Methanotrophs belong to the Alpha- or Gamma-classes of *Proteobacteria,* as well as to the phylum *Verrucomicrobia.* Only few representatives of alphaproteobacterial methanotrophs can utilize a limited range of polycarbon compounds and none of the methanotrophs can use sugars as growth substrates^1–3^. Nevertheless, bioinformatics and biochemical studies have shown that at least three pathways for cleavage of hexose phosphates are operative in gammaproteobacterial (Type I) methanotrophs: the modified pyrophosphate-dependent glycolysis, the Entner–Doudoroff pathway and the oxidative pentose phosphate cycle; the phosphoketolase glycolytic pathway is also encoded by the methanotrophs^4^. These routes start from hexose phosphates which are the first metabolites of the ribulose monophosphate (RuMP) pathway for C1 assimilation and lead to the formation of C2, C3 and C4 compounds, as well as to the redox cofactors NAD(P)H and ATP generation^5,6^. Moreover, the enzymes for glucose and fructose phosphorylation (ATP-glucokinase and ATP-fructokinase and, additionally, polyphosphate-dependent glucokinase in several species) have been characterized in Type I methanotrophs^7–9^. In spite of such a high versatility of carbohydrate metabolism, Type I methanotrophs demonstrate obligate dependence on C1 compounds and the biochemical basis of their inability to grow on polycarbon compounds remains unclear. Moreover, the genomic analysis also indicates that some Type I methanotrophs encode the putative glucose-1-dehydrogenase (GDH) and gluconate kinase (GntK), whose functionality still has to be elucidated (Fig. 1).

Glucose 1-dehydrogenases are the cofactor-dependent enzymes catalyzing oxidation of the first hydroxyl group of D-glucose to form D-glucono-1,5-lactone. The bacterial GDH (EC1.1.1.47) that catalyzes β-D-glucose oxidation, simultaneously converting NAD(P)^+^ to NAD(P)H, belongs to the extended superfamily of short-chain dehydrogenases/reductases (SDR)^10^. The enzymes of this family show the typical GXGXXG⁄A fingerprint motif found in most NADP^+^-binding proteins^11,12^. Then D-glucono-1,5-lactone undergoes spontaneous hydrolysis in water (or by gluconolactonase) to gluconic acid, and the latter can be further phosphorylated to 6-phosphogluconate by gluconokinase (GntK) (EC 2.7.1.12). NADP^+^-GDHs have been described in gram-positive heterotrophic bacteria and archaea^13^. In archaea, however, GDH reaction product is further converted to 2-keto-3-deoxygluconate by gluconate dehydratase^14^. The Entner–Doudoroff pathway, which includes glucose dehydrogenase and gluconokinase, in addition to glycolysis and the oxidative pentose phosphate pathway, has been found in plants and cyanobacteria, and simultaneously anabolic (Calvin-Benson-Basham cycle) and catabolic processes (glycolytic pathways) have been demonstrated^15,16^.

This paper reports for the first time on the characteristics of NAD(P)^+^-dependent glucose dehydrogenase and gluconokinase in gram-negative bacteria unable to use carbohydrates as growth substrates. Characterization of the mutants with knockout of the enzymes leading to 6-phosphogluconate from free glucose, as well as bioinformatics analysis, have shown the in vivo functioning of the gluconate branch linking glycogen metabolism and the Entner–Doudoroff pathway in obligate methanotrophs.

## Results

### GDH-His_6_ expression, purification and structure

The sequences encoding the proteins with ~ 39% identity to that of GDH characterized in *Bacillus subtilis* (BSU02830) were found in the *Mtm. alcaliphilum* and *Mb. luteus* genomes. The full sequences of the respective ORFs (CCE23386 and WP_027157932) were expressed in *E. coli* Rosetta (DE3). The recombinant proteins, MtmGDH and MbGDH with 6 histidines at the N-termini, were purified by one-step affinity metal-chelating chromatography. SDS-PAGE of each enzyme revealed one band corresponding to a molecular mass of ~ 30 kDa, which is in accordance with the theoretically calculated masses of 28.6 kDa and 29.1 kDa for GDH from *Mtm. alcaliphilum* or *Mb. luteus*, respectively. Gel filtration of the purified enzymes indicated a tetrameric structure of both methanotrophic GDHs (with Mr 112 kDa for MtmGDH and 100 kDa for MbGDH) (Fig. S1).

### Catalytic properties of GDHs

**Figure 2:**
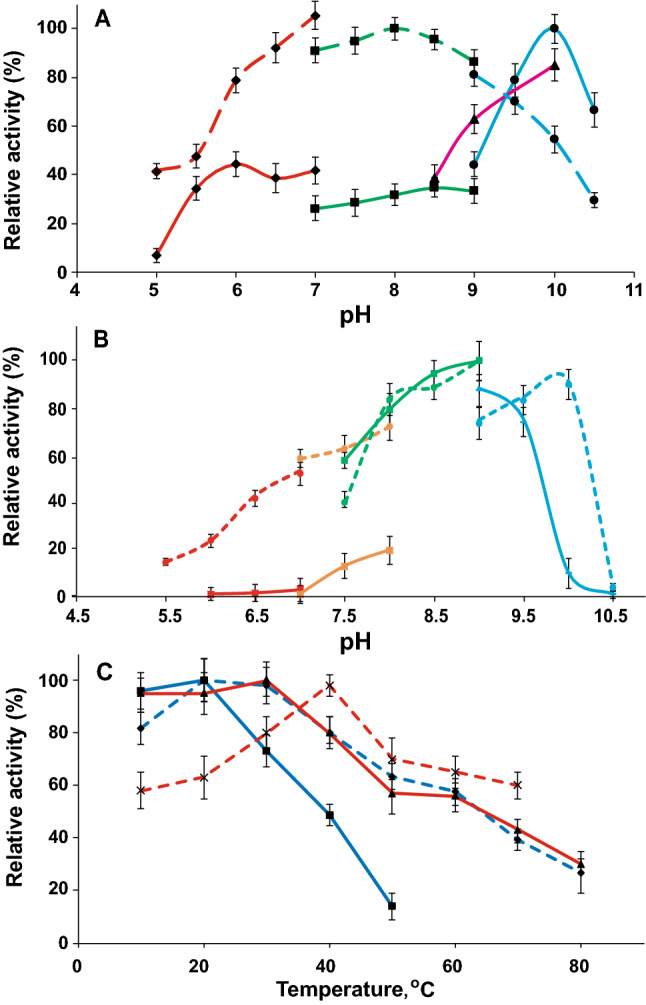
The effect of pH on the activity of GDHs (**A**) and GntKs (**B**) from *Mtm. alcaliphilum* (solid line) and *Mb. luteus* (dashed line). The following buffers were used for activity measurement: MES-NaOH (red line), Tris–HCl (green line), K-P buffer (orange line), CHES–NaOH (purple line), and Glycine–NaOH (blue line). 100% activity of GDH corresponds to 90 U/mg protein for *Mtm. alcaliphilum* GDH and 28 U/mg protein for *Mb. luteus* GDH; 100% activity of GntK corresponds to 20 and 56 U/mg protein for *Mtm. alcaliphilum* and *Mb. luteus* GntK, respectively. (**C**) The effect of temperature on the activity of GDHs (dark blue line) and GntK (red line) from *Mtm. alcaliphilum* (solid line) and *Mb. luteus* (dashed line).

Both recombinant GDHs catalyzed the D-glucose-dependent reduction of NAD^+^ or NADP^+^, but they were inactive with other sugars tested (L-glucose, fructose, mannose, galactose, sorbose and N-acetyl-glucosamine). The enzyme from *Mtm. alcaliphilum* was most active at pH 10.0 (Fig. 2A) and 10–20 °C (Fig. 2C). It lost 52% or 87% of activity after 1-h exposure at 40 °C and 50 °C, respectively. 50 mM NH_4_Cl stimulated the enzyme activity 1.5-fold (Supplementary Table S1). In the presence of 0.2 M NaCl or 2 M KCl, the enzyme activity decreased by 50% and 88%, respectively, but chlorides of bivalent metals had no significant effect. The dependence of activity on the concentrations of substrates obeyed the Michaelis–Menten kinetics. The maximum reaction rate with NAD^+^ was higher than with NADP^+^ (95 versus 39 U/mg protein) (Table 1). The apparent *K*_m_ values measured at 30 °C and pH 10 were as follows: 92 ± 4 mM for glucose, 0.36 ± 0.02 mM for NAD^+^, and 0.15 ± 0.01 mM for NADP^+^. For testing of influence of histidines on the kinetic parameters of MtmGDH, we carried out the cutting off His_6_-tags and found that the apparent *K*_m_ values for the substrates did not change compared to the His_6_-tagged enzyme (Table 1).

**Table 1:** The kinetic properties of GDHs from methanotrophs.

Parameter	*Mtm. alcaliphilum*		*Mb. luteus*
	His_6_-tag at the N-terminus	Without His_6_-tag	
M.m. (number of subunits), kDa	112 (29 × 4)		100 (29 × 4)
*V*_max_, U/mg protein (NAD^+^)	95 ± 3	ND	31 ± 2
*V*_max_, U/mg protein (NADP^+^)	39 ± 2	ND	43 ± 6
***K***_**m**_**, mM:**			
Glucose	92 ± 4	86 ± 1	16 ± 1
NAD^+^	0.36 ± 0.02	0.39 ± 0.06	0.12 ± 0.02
NADP^+^	0.14 ± 0.01	0.34 ± 0.02	1.6 ± 0.3
*k*_cat NAD,_ 1/min	10.6	ND	3
*k*_cat NADP,_ 1/min	0.35	ND	4
*k*_cat_/*K*_m_^glucose^, 1/(mM × min)	0.1	ND	0.2
*k*_cat_/*K*_m_^NAD^, 1/(mM × min)	30	ND	26
*k*_cat_/*K*_m_^NADP^, 1/(mM × min)	2.5	ND	2.7
Specificity (*k*_*cat*_*/K*_m_)^NAD^/(*k*_*cat*_*/K*_m_)^NADP^	12	ND	10

*ND *not determined.

GDH from *Mb. luteus* was most active at pH 8.0 (Fig. 2A) and 20–30 °C (Fig. 2C). The enzyme lost 37% and 61% of the activity after heating for 1 h at 50 °C and 70 °C, respectively. The activity slightly increased in the presence of 50 mM KCl, NaCl or NH_4_Cl. However, in the presence of 0.25 M NaCl or 2 M KCl, the residual activity was 84 and 39%, respectively. The chlorides of Ni^2+^, Cd^2+^, Zn^2+^ and Co^2+^ completely inhibited *Mb. luteus* GDH (Supplementary Table S1). The maximum of activity with NADP^+^ was higher than with NAD^+^ (43 vs 31 U/mg protein) (Table 1). MbGDH had a higher affinity for glucose compared to the *Mtm. alcaliphilum* enzyme (*K*_m_ 16 mM vs. 92 mM) but a lower affinity for NADP^+^ (*K*_m_ 1.6 mM vs. 0.01 mM) (Table 1). The calculated catalytic constants *k*_cat_ and *k*_cat_/*K*_m_ showed that both enzymes had low specificity for glucose and exhibited an order of magnitude higher specificity for NAD^+^ than for NADP^+^ (Table 1). The activities of both GDHs did not significantly vary in the presence of various organic metabolites (Supplementary Table S2). The activity of MtmGDH slightly increased (by 40%) in the presence of 1 mM isocitrate, and the activity of *Mb. luteus* GDH decreased by 40% in the presence of 10 mM glucose-6-phosphate.

### Expression, purification and structure of GntK-His_6_ from methanotrophs

The genes encoding the gluconate kinase from *Mtm. alcaliphilum* (CE23379) and *Mb. luteus* (WP_051299846) were heterologously expressed in *E. coli* Rosetta (DE3). The recombinant proteins MtmGntK and MbGntK with 6 histidines at the C-terminus were purified by affinity Ni-NTA chromatography. SDS-PAGE of each enzyme showed one band corresponding to a molecular mass of ~ 20 kDa, which is consistent with the theoretically calculated masses. Gel filtration of the purified enzymes indicated a monomeric structure of both GntKs (Fig. S1).

### Catalytic properties of GntKs from methanotrophs

The recombinant MtmGntK demonstrated activity 26 U/mg protein. The enzyme was active in the pH range from pH 7.5 to pH 9.5 with the optimum at pH 9.0 (Fig. 2B) and in the temperature range from 10 to 30 °C (Fig. 2C). It was quite stable, withstanding 2-h heating at 30–50 °C without a decrease in activity. Nonetheless, its activity was fully lost after 5-min exposure at 70 °C. No allosteric regulators were found for MtmGntk; however, various metals significantly reduced the activity (Supplementary Tables S1, S3). Thus, CuCl_2_, CdCl_2_, NiCl_2_, ZnCl_2_ and BaCl_2_ at a concentration of 1 mM almost fully inhibited the activity, while CaCl_2_ and CoCl_2_ decreased the activity by 60 and 70%, respectively. 50 mM NH_4_Cl and KCl reduced the activity by 30%. 50 mM NaCl had no influence on the enzyme activity whereas in the presence of 0.5 M NaCl the activity of MtmGntK decreased by 80%. The dependence of the MtmGntK activity on gluconate concentration obeyed the Michaelis–Menten kinetics: the apparent *K*_m_ value was 0.60 ± 0.03 mM (Table 2). In response to the changes in ATP concentration, the enzyme behavior did not obey the Michaelis–Menten kinetics: the *S*_0.5_ value, 1.3 ± 0.2 mM; the Hill coefficient, *n* = 1.6.

**Table 2:** The kinetic properties of GntKs from methanotrophs.

Parameter	*Mtm. alcaliphilum*	*Mb. luteus*
M.m. (number of subunits), kDa	20 (1)	20 (1)
*V*_max_, U/mg protein	25.8 ± 0.4	56.1 ± 0.9
***K***_**m**_ **or S**_0.5_*, mM:		
Gluconate	0.6 ± 0.04	0.24 ± 0.01
ATP	1.3 ± 0.2*(n = 1.6)	0.042 ± 0.002* (n = 2.3)
*k*_cat_^Gluconate^_,_ 1/min	0.26	0.77
*k*_cat_^ATP^_,_ 1/min	0.52	1.1
*k*_cat_/*K*_m_^gluconate^, 1/(mM × min)	0.4	3.2
*k*_cat_/*K*_m_^ATP^, 1/(mM × min)	0.4	26

The maximal activity of MbGntK under optimal conditions (pH 9.0, 40 °C) was 56 U/mg protein (Fig. 2). Unlike MtmGntK, the enzyme from *Mb. luteus* fully lost the activity after 5-min exposure at 50 °C, while the activity did not change after 2-h exposure at 30 °C. No allosteric regulator of the enzyme was found. CuCl_2_ almost completely suppressed the MbGntK activity, while CdCl_2_ and ZnCl_2_ reduced its activity by 80 and 70%, respectively (Supplementary Table S1). The activity slightly increased in the presence of monovalent cations Na^+^ and NH_4_^+^ (50 mM); however, in the presence of 0.5 or 1 M NaCl, the activity decreased by 70 or 10%, respectively. The apparent *K*_m_ value for gluconate was 0.23 ± 0.01 mM. Similarly to the enzyme from *Mtm. alcaliphilum*, the dependence of MbGntK activity on ATP concentration did not obey the Michaelis–Menten kinetics; *S*_0.5_ was 0.042 ± 0.002 mM, the Hill coefficient *n* = 2.3.

### The activities of GDH and Glk in cell-free extracts of methanotrophs

The activity of GDH in cell-free extracts of *Mtm. alcaliphilum* was found to be ~ 20 mU/mg protein in the presence of 200 mM glucose in the reaction mixture, but the reaction quickly stopped if 20 mM glucose concentration was used to measure the activity. It corresponded with the extraordinary high apparent *K*_m_ value (92 mM) found for the purified enzyme. This implies the in vivo expression of glucose dehydrogenase in *Mtm. alcaliphilum.* The specific activity of GDH in the cell-free extracts of *Mb. luteus* in the presence of 200 mM glucose was ~ 28 mU/mg protein; at 20 mM glucose, it was ~ 14 mU/mg protein.

The specific activity of glucokinase (Glk) in cell-free extracts of *Mtm. alcaliphilum* was ~ 30 mU/mg protein. In response to the increase of glucose concentration from 5 to 200 mM in the reaction mixture, the enzyme activity increased to 60 ± 5 mU/mg protein. This increase can be explained by appearance of the low affinity GDH.

### The analysis of activities of the putative promoter regions of the *gntk* and *amy* genes

We were unsuccessful to find activity of gluconate kinase in the cell-free extracts from both methanotrophs. Therefore, using *cat* as a reporter gene (encoding chloramphenicol acetyltransferase), we analyzed the activities of the putative promoter region of the *gntk* gene in *Mtm. alcaliphilum*. In cell-free extracts of *Mtm. alcaliphilum* pMHA:P*gntk*-*cat* grown under methane or in the presence of methanol, the activities of chloramphenicol acetyltransferase were 2.6 ± 0.8 and 5.1 ± 0.3 mU/mg, respectively.

In *Mtm. alcaliphilum,* 10 bp upstream the *gntk* gene the *amy* gene encoding alpha amylase is located (Fig. S2). The activity of the *amy* promoter was also analyzed. When *Mtm. alcaliphilum* pMHA:P*amy*-*cat* was grown on methanol or under methane, the activities of chloramphenicol acetyltransferase were found (9.7 ± 0.5 or 6.0 ± 0.3 mU/mg respectively). Consequently, one may expect that the *gntk* and *amy* genes are functional.

### Phenotypic characterization of insertional mutants

To prove the in vivo glucose metabolism through the GDH/GntK reactions in *Mtm. alcaliphilum*, the strain with insertion in the *gdh* gene was obtained but changes in growth characteristics in the mutant strain were not identified. This can be due to the operation of glucokinase highly specific to glucose (*K*_m_ 0.32 mM)^8^. In the double mutated strain *gntk*^*─*^*/glk*^*─*^, the GDH activity was 22 ± 5 mU/mg protein but Glk activity was not detected. The *gdh*^*─*^*/glk*^*─*^ strain lacked both GDH and Glk activities.

**Figure 3:**
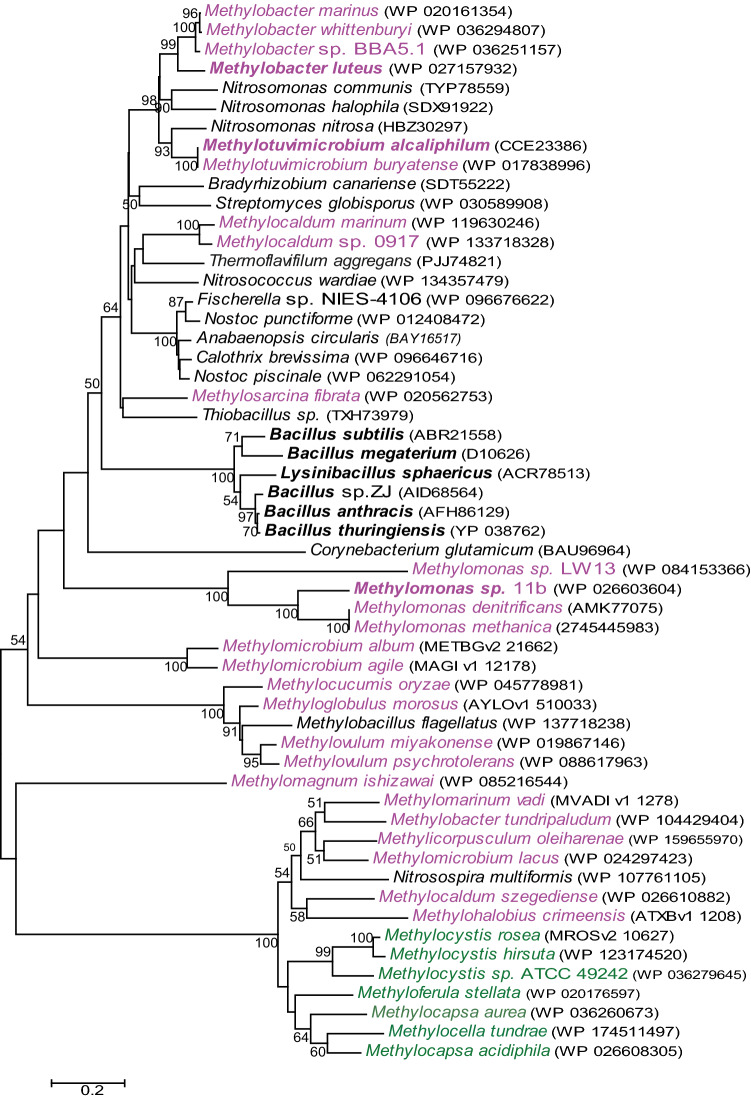
The phylogenetic tree built of the amino acid sequences of various putative and characterized bacterial NAD(P)^+^-dependent glucose dehydrogenases. The characterized enzymes are in bold: *Bacillus thuringiensis*^17^, *Bacillus* sp. ZJ^18^, *Bacillus megaterium*^19^, *Bacillus subtilis*^20^ and *Lysinibacillus sphaericus*^21^. The amino acid accession numbers in the Gene Bank are in brackets. Gammaproteobacterial methanotrophs are purple colored, alphaproteobacterial methanotrophs are green colored. The scale bar corresponds to the number of substitutions per site. The sequences from NCBI, IMG/MER and MicroScope databases (http://www.ncbi.nlm.nih.gov, https://mage.genoscope.cns.fr/, https://img.jgi.doe.gov/) were obtained by BLAST searches. The alignments of amino acid sequences and the phylogenetic analysis were performed using MEGA 6 and the Neighbor-Joining model^22^.

In the presence of methane or methanol, the double mutation strains grew 1.5 times slower compared to the wild type (WT) strain 20Z (Fig. 3S). The same effect was observed in a single *glk*^*─*^ mutant^8^. In contrast to the *glk*^─^ strain, where the content of intracellular glucose did not actually differ from the WT strain^8^, the intracellular pools of glucose in the *gdh*^*─*^*/glk*^*─*^ strain grown on methane or methanol increased 50- and 32-fold, respectively (Table 3). At the same time, the glucose content in cells of the *gntk*^*─*^*/glk*^*─*^ strain increased only 3–4 times regardless of the growth substrate. Only a slight increase in glucose in the *gntk*^*─*^*/glk*^─^ mutant indicated the functioning of GDH, which converts excess glucose into gluconate. Another evidence of the functionality of GDH and GntK enzymes is accumulation of gluconic acid in the *gntk*^*─*^*/glk*^*─*^ strain (Table 3). In cells of these mutants grown in either methane or methanol, glycogen accumulation dramatically decreased (Table 3). It should be noted that glycogen concentration in the methane grown *gdh*^*─*^*/glk*^*─*^ strain dropped 690 times compared to the WT strain, whereas a minor glycogen decrease was observed in the methanol-grown *gntk*^*─*^*/glk*^*─*^ strain (~ 6 times). The *gntk*^*─*^*/glk*^*─*^ strain growing on methanol accumulated the highest level of intracellular trehalose, but least of all when growing under methane (Table 3). In cells of the *gntk*^*─*^*/glk*^*─*^ and *gdh*^*─*^*/glk*^*─*^ strains growing on methanol a mixture of malto-derivatives of carbohydrates were detected, but it was very difficult to identify these compounds (the individual components of the mixture) by the H^1^-NMR method.

**Table 3:** Accumulation of carbohydrates (mg per g of DCW) in the wild type (WT) cells of *Mtm. alcaliphilum* and mutant strains deficient in glucose dehydrogenase/glucokinase (*gdh*^-^/*glk*^-^) and gluconate kinase/glucokinase (*gntk*^-^/*glk*^-^). *ND*, not detected.

Metabolite Growth substrate	Sucrose	Glycogen	Glucose	Gluconate	Trehalose	Glucose-1P	Glucose-6P
**WT**							
Methane	17.3 ± 0.9	138 ± 8	0.14 ± 0.04	ND	0.04 ± 0.01	0.12 ± 0.02	0.09 ± 0.01
Methanol	12.2 ± 0.3	322 ± 9	1.27 ± 0.03	ND	0.06 ± 0.01	0.10 ± 0.06	0.18 ± 0.03
***gdh***^**-**^**/glk**^-^							
Methane	4.9 ± 1.2	0.20 ± 0.04	7.3 ± 1.1	ND	0.56 ± 0.16	0.12 ± 0.01	0.13 ± 0.08
Methanol	0.82 ± 0.08	4.9 ± 0.5	32.2 ± 1.4	ND	0.83 ± 0.11	0.26 ± 0.11	0.41 ± 0.03
***gntk***^**-**^**/glk**^-^							
Methane	8.7 ± 2.0	1.24 ± 0.06	0.41 ± 0.04	0.83 ± 0.14	0.16 ± 0.1	0.14 ± 0.04	0.15 ± 0.01
Methanol	7.6 ± 1.2	56 ± 5	5.3 ± 1.1	1.30 ± 0.23	1.84 ± 0.18	0.24 ± 0.11	0.2 ± 0.02

The samples for analyses were drawn at late exponential phase of the culture growth.

### The sequence and distribution of GDH in methanotrophs

As can be judged from protein sequences, GDHs from *Mtm. alcaliphilum* and *Mb. luteus* belong to the superfamily of short-chain dehydrogenases/reductases (SDR). These enzymes show the typical GXGXXG fingerprint motif found in NADP^+^-binding proteins^11,12^ (Fig. 4S). There are some differences in the composition of amino acids forming the active center in GDH from *Bacillus megaterium* and those from *Mtm. alcaliphilum* and *Mb. luteus* (marked * in Fig. 4S). The amino acids at the C-terminus of *B. megaterium* GDH (Q257, A258, R260 and G261) directly interact with glucose and thus determine the kinetic properties of the enzyme. The C-termini at GDHs from *Mtm. alcaliphilum* and *Mb. luteus* are significantly different from the C-terminus of the *B. megaterium* enzyme, which may be reflected in the kinetic properties of methanotrophic enzymes (Fig. 4S). The only difference in amino acids forming hydrogen bonds with the hydroxyl groups of glucose between the *Mtm. alcaliphilum* and *Mb. luteus* GDHs is the replacement of aspartate (D219) by glutamate (E219). The GDHs from other methanotrophs have more differences in amino acids presumably forming the active center and interacting with glucose (Fig. 4S).

Analysis of the genome databases available showed that only a number of methanotrophs have GDH homologues (Fig. 3, Supplementary Table S4). GDH homologs are present in some other Type I methanotrophs: *Methylosarcina fibrata* (60% identity of translated amino acids to MtmGDH) and some representatives of the genera *Methylocaldum* (62% identity) and *Methylomonas* (29% identity) (Fig. 3). However, the genomes of *Methylomonas* and *Methylosarcina* have no gene for gluconate kinase. At the same time, gluconate kinases from *Mtm. alcaliphilum, Methylocaldum szegediense* and *Methylocella silvestris* have only 32–37% identity with each other.

A number of other methanotrophs have putative GDHs, which have very low similarity (14–27% identity) to *Mtm. alcaliphilum* and *Bacillus megaterium* enzymes, but belong to the SDR family (Fig. 3).

## Discussion

In this study we have characterized for the first time glucose dehydrogenase and gluconate kinase, the starting enzymes for glucose metabolism, in aerobic methanotrophs, bacteria which unable to use sugars as growth substrates. The GDHs from *Mtm. alcaliphilum* and *Mb. luteus* are strongly specific for D-glucose and use NAD^+^ as the preferred cofactor. To date, microbial NAD(P)^+^-GDHs have been characterized in gram-positive bacteria, cyanobacteria, archaea, fungi and yeasts, as well as in plants, whereas GDHs investigated in gram-negative bacteria were PQQ-dependent^23^. GDHs from *Bacilli* can oxidize a broad range of substrates^24^. The NAD(P)^+^-GDH from *B. thuringiensis* oxidizes glucose (*K*_m_ 14 mM) and 2-deoxy-D-glucose (*K*_m_ 12.2 mM)^17^, and the enzyme from *Lysinibacillus sphaericus* also oxidizes disaccharides such as maltose, lactose, and sucrose^21^. GDHs from the archaea *Thermoproteus tenaxis* and *Sulfolobus solfataricus* have *K*_m_ 0.3–0.4 mM for glucose, *K*_m_ values for the enzymes from bacteria utilizing the sugar as growth substrate are in a wide range (0.5–42 mM)^23,25^ and *K*_m_ of the enzyme from *Mb. luteus* is within the same range. Curiously, GDH from *Mtm. alcaliphilum* remains the absolute record holder for the highest *K*_m_ (92 mM). Similarly to the GDHs from *Bacillus* sp. ZJ and *L. sphaericus*^18,21^, methanotrophic GDHs are tetramers.

Previously, a single *glk*^*─*^ mutant exhibiting a drastic decrease in glycogen level (50 times compared to the WT strain) has been characterized while its intracellular glucose level remained almost unaltered^8^. In this work, the *gdh*^*─*^*/glk*^*─*^ mutant lacking genes for both enzymes responsible for activation of free glucose demonstrated considerable increase in intracellular glucose content (Table 3). This indicated that, despite the high *K*_m_, GDH in *Mtm. alcaliphilum* is involved in maintaining the intracellular pool of glucose. Only a slight increase of glucose content in the *gntk*^*─*^*/glk*^*─*^ strain additionally confirmed participation of GDH in utilization of the sugar. The occurrence of gluconate in the *gntk*^*─*^*/glk*^*─*^ strain where the gene for gluconate kinase is turned off and GDH working, proves the in vivo participation of both enzymes in free glucose removal.

In cells of the *glk*^*─*^, *gdh*^*─*^*/glk*^*─*^ and *gntk*^*─*^*/glk*^*─*^ strains, trehalose level was drastically increased as compared to the WT cells. The genomic analysis allows us to propose that only glycogen can be a source of trehalose. Thus, the genome of *Mtm. alcaliphilum* harbors the *treY* and *treZ* genes encoding putative maltooligosyltrehalose synthase (CCE23905) and trehalohydrolase (CCE23904) which convert the terminal unit of the linear maltodextrin (glycogen or starch) into trehalose via maltooligosyltrehalose (Fig. 3). It also has the gene for putative maltose alpha-D-glucosyltransferase (trehalose synthase, TreS, CCE22655) which catalyzes the reversible conversion of maltose into trehalose as described for the first time in mycobacteria^26^. At the same time, *Mtm. alcaliphilum* lacks the *otsA-otsB* genes encoding trehalose 6-phosphate synthase and trehalose 6-phosphate phosphatase responsible for trehalose synthesis at many (micro)organisms. The methanotroph has gene encoding 4-alpha-glucanotransferase (MalQ, CCE25166) which recognizes maltose and longer maltodextrins and preferentially removes glucose from the reducing ends of maltose and from small maltodextrins and transfers the remaining enzyme-bound dextrinyl residue to the non-reducing ends of other maltodextrins, thus forming longer maltodextrin chains^27^. Two Glg enzymes are essential for endogenous maltotriose formation in *Mtm. alcaliphilum*: glycogen phosphorylase GlgP (CCE22814; CCE22130; CCE25654) which shortens the branch chains of glycogen to the lengths of maltotetraosyl and maltotriosyl units, and GlgX (CCE25166), which releases the branch chains. Some GlgPs can act as maltodextrin phosphorylases MalP^28^ (Fig. 1). Therefore, glycogen serves as the main source of free glucose for GDH and Glk reactions. Since glucose and trehalose were found in wild type cells (Table 3), the process of glycogen degradation in *Mtm. alcaliphilum* most likely goes along with glycogen synthesis. The concerted action of glycogen synthesizing and degrading enzymes has been recently revealed in *Corynebacterium glutamicum* and *E. coli* ^29^. The simultaneously occurring glycolysis and the oxidative pentose phosphate pathway in addition to the Entner–Doudoroff pathway shunt including glucose dehydrogenase and gluconate kinase has been proven for plants and cyanobacteria^15,16,30^.

In *Mtm*. *alcaliphilum* 20Z, glucose derived from glycogen degradation is utilized mainly by glucokinase due to its higher specificity to the sugar than GDH. The glucose 6-phosphate formed in Glk reaction is further converted by phosphoglucose isomerase into fructose 6-phosphate, which enters glycolysis after phosphorylation by PPi-dependent phosphofructokinase and/or the Entner–Doudoroff pathway via glucose 6-phosphate dehydrogenase. In the case of glycogen decomposition and/or inhibition of glucokinase activity by ADP^8^ (*K*_i_ 2.34 mM), GDH can be involved in the glucose utilization. It should be noted that 6-phosphogluconate is a substrate for 6-phosphogluconate dehydrogenase reaction which produces ribulose 5-phosphate, an acceptor of formaldehyde in the key reaction of carbon assimilation catalyzed by hexulosephosphate synthase (Fig. 1). Therefore, metabolism of glucose via the gluconate shunt can be an anaplerotic mechanism that replenishes the RuMP cycle by tapping cellular carbohydrate reservoirs under fluctuations in content of carbon substrate in the environment.

The ability of these bacteria to obtain the key central metabolites from glycogen could allow them to survive the lack of the growth substrate in the environment at the expense of their own internal resources. Previous studies have shown that thermotolerant methanotroph *Methylococcus* strain NCIB 11083 can metabolize glycogen under aerobic starvation conditions, and this process could generate sufficient energy for protein synthesis in the absence of methane^31^. Recently we have shown that inactivation of the genes encoding the key enzymes of sucrose and glycogen synthesis reduced growth rate of *Mtm. alcaliphilum* mutant on methane or methanol and increased the protein content in cells of the methanotroph^32^.

The reasons for the inhibition of growth of the strains lacking the genes for metabolism of free glucose are still unclear. Apparently, the accumulation of glucose in the cytoplasm and dramatic drop in glycogen level in the mutants indicate a permanent process of decomposition of glycogen, and inhibition of the polymer synthesis (Fig. S5). The molecular mechanisms underlying the regulation of the synthesis and degradation of the storage polymer in bacteria remain to be elucidated. A direct non-enzymatic effect of glucose on bacterial proteins/enzymes and the triggering of stress responses are not excluded^33^.

The genomic analyses showed that all Type I methanotrophs possess genes encoding glycogen synthase (Supplementary Table 4S). They also harbor up to two genes for glucokinases with a single exception *Methylocaldum marinum* S8 lacking Glk encoding gene. None of them encode PTS system for glucose transport. The GDH homologues present in Type I methanotrophs (usually along with GntK ones) with exceptions of *Methylococcus capsulatus* (Bath and Texas), *Methylomonas methanica* MC09, *Methylomonas* sp. MK1 and *Methylotuvimicrobium kenyense* AMO1 lacking both gluconate shunt enzymes. Several species, for example *Methylosarcina fibrata* AML-C10 and *Methylomicrobium album* BG8 encode gluconate dehydrogenase (GntD) instead of the GntK thuds implying another variant of non-phosphorylated gluconate shunt (Supplementary Table 4S).

Among Type II methanotrophs, synthesizing hexosephosphates mainly through gluconeogenesis, almost all representatives have GlgA encoding genes (Supplementary Table 4S). The exceptions are all *Methylosinus* strains and *Methylocystis* sp. LW5 lacking *glgA* genes. About half of the genomes of this group methanotrophs sequenced today also code GDH and few species also have Glk encoding genes. All Type II methanotrophs possess genes for PTS system for sugar transport. Thus, genomic analysis demonstrates high versatility of carbohydrate metabolism in methanotrophs, the bacteria unable to grow on any sugars. The redundancy of the pathways of sugar metabolism in obligate methanotrophs determines their ability to survive under the conditions of CH_4_ deficiency, but the question about the causes of their inability to use glucose as a carbon and energy source for growth is still open.

## Materials and Methods

### Bacteria and growth conditions

*Mb. luteus* (IMV-B-3098) and *Mtm. alcaliphilum* 20Z (VKM B-2133^T^ = NCIMB 14124^T^) were grown at 30 °C on a nitrate salt medium. For the growth of *Mtm. alcaliphilum* 20Z, the medium additionally contained 0.1 M NaHCO_3_ and 0.3 M NaCl^34^. Methane (50% in the gas phase) or methanol (0.5%, v/v) was used as a carbon source. *Escherichia coli* Rosetta (DE3) obtained from Stratagene (La Jolla) was grown at 37 °C on a Luria–Bertani (LB) agar or in broth. Kanamycin (50 μg/mL) and/or chloramphenicol (25 μg/mL) was added to the growth medium as necessary.

### Cloning, preparation and purification of the recombinant enzymes

To amplify the gene encoding ubiquitin with 6 histidines at the N-terminus from the pHUE plasmid (Supplementary Table S5), the Ub-F/Ub-R primers (Supplementary Table S6) and vector pET28 for cloning were used. The *gdh* gene was amplified by PCR from the DNAs of *Mtm. alcaliphilum* or *Mb. luteus* using the primers presented in the Supplementary Table S4. The purified PCR product corresponding to the *Mtmgdh* gene was ligated into the pET28 or pET28Ub-shHis vectors at the NdeI/HindIII or SacII/HindIII restriction sites, respectively. The PCR product carrying the *Mbgdh* gene was ligated into pET28Ub-shHis at the SacII/HindIII restriction sites. The resulting vectors pET28:gdhMtm, pET28Ub-shHis:gdhMtm, or pET28Ub-shHis:gdhMb were transferred into *E. coli* Rosetta (DE3), enzyme synthesis was induced by 0.5 mM isopropyl-β-D-thiogalactopyranoside, and cells were incubated for 15 h at 18 °C. Recombinant enzymes with His_6_ at the N-termini were purified as described^35^. To remove His-tags, the enzymes were incubated for 18 h with Usp2-His_6_ protease (50 μg of protease per 1 mg of protein) at + 4 °C. The efficiency and completeness of hydrolysis were checked by SDS-PAAG electrophoresis. The final purification of GDH preparations from the cut-off His-tag and Usp2-His6 were not carried out, since GDH activity was lost during dialysis.

The vector pET30 (Novagen) was used to amplify the *gntk* genes from methanotrophic DNAs by the respective primers (Supplementary Table S6). The resulting vector pET30:gntkMtm or pET30:gntkMb was transferred into *E. coli* Rosetta (DE3). The purification of the recombinant GntKs was carried out as described above. Purified recombinant enzymes were stored in 40% glycerol at −20 °C.

### Determination of molecular weight of recombinant enzymes

Gel filtration was performed on the XK 16/100 Superdex 200 column (GE Healthcare) balanced with 0.02 M Tris–HCl (pH 7.0) containing 0.5 M NaCl. The following protein markers (Sigma-Aldrich) were used to build the calibration curve: carbonic anhydrase (29 kDa), albumin (66 kDa), alcohol dehydrogenase (150 kDa), β-amylase (200 kDa), apoferritin (443 kDa). GDHs with the cut-off His-tag were used to determine molecular weights of the enzymes.

### Assay of enzyme activities

#### GDH activity assay

The activity of GDH from *Mtm. alcaliphilum* was measured by recording the rate of NAD^+^ or NADP^+^ reduction at 30 °C. 1 mL of the reaction mixture routinely contained 50 mM Glycine–NaOH buffer, pH 10.0; 0.3 mM NAD(P)^+^ and ~ 1 μg of GDH. The reaction was started with 200 mM D-glucose. The activity of *Mb. luteus* GDH was measured using 20 mM D-glucose in 50 mM Tris–HCl, pH 8.5. Ribose, trehalose, deoxyglucose, galactose, fructose, arabinose, xylose, acetyl-glucosamine, maltose, sorbose, mannitol, mannose, sucrose, lactose and L-glucose at a final concentration of 10 mM were tested as potential substrates for glucose dehydrogenases. The effect of pH on the activity was studied using the following buffers (50 mM): Glycine–NaOH (pH 9.0–10.5), CHES-NaOH (pH 8.5–10.0), Tris–HCl (pH 7.5–9.0), and MES-NaOH (pH 5.0–7.0). KCl, NH_4_Cl or NaCl at a final concentration of 50 mM were added to test the effect of monovalent ions on the enzyme activity. The effects of divalent metals were assessed using MgCl_2_, MnCl_2_, CoCl_2_, CuCl_2_, CdCl_*2*_, NiCl_2_ or ZnCl_*2*_ at a final concentration of 1 mM. Glucose-6-phosphate, glucose-1-phosphate, fructose-6-phosphate, fructose-1,6-bisphosphate (5 mM), pyruvate, PEP, oxaloacetate, lactate, malate, isocitrate, citrate, succinate, serine, α-ketoglutarate, ATP, ADP, AMP and PPi (1 mM) were examined as potential inhibitors or activators. To determine the optimal temperature for enzyme activity, the reaction was carried out in a temperature range of 10–70 °C. To determine the apparent *K*_m_ values, substrate concentrations in the ranges of 0.391–400 mM (glucose), 0.0078–1 mM (NADP^+^), 0.0078–0.75 mM (NAD^+^) were used to measure the activity.

#### GntK activity assay

The activity of GntK was measured in the reaction mixture containing 50 mM Tris–HCl buffer, pH 9.0; 8 mM MgCl_2_; 6 mM gluconate; 5 mM ATP; 0.3 mM NADP^+^; 5U 6-phosphogluconate dehydrogenase (6PGDH, Sigma-Aldrich) as a coupling enzyme and ~ 15 µg GntK. The activity of *Mb. luteus* GntK was assayed in 50 mM Tris–HCl buffer (pH 8.0). The reaction was started with 6 mM sodium gluconate. To study the effect of pH, the following buffers were used (50 mM): Carbonate (pH 9.0–10.5), TAPS-NaOH (pH 8.0–9.0), Tris–HCl (pH 7.5–9.0), K-phosphate (pH 7.0–8.0), MES-NaOH (pH 5.0–6.0) or citrate (pH 4.0–5.0). Glucose 6-phosphate, glucose 1-phosphate, 6-phosphogluconate, PEP, ADP (5 mM at the final concentration), AMP (4 mM), pyruvate (3 mM), PPi (2 mM), oxaloacetate and α-ketoglutarate (1 mM) were tested as potential inhibitors or activators. To determine the apparent *K*_m_, the activity was measured in the concentration ranges of 0.031–8 mM gluconate and 0.015–4 mM ATP. The NAD^+^/NADP^+^ reduction rates were measured at 340 nm with a UV-1700 spectrophotometer (Shimadzu). The apparent *K*_m_ and *V*_max_ values were calculated using Sigma Plot (version 10). The exponentially grown cells were used to measure enzymatic activity in cell-free extracts.

### Mutant generation

The strains of *Mtm. alcaliphilum* with the double *gdh/glk* and *gntk/glk* gene mutations were generated by insertion mutagenesis. Intergenic regions flanking the *gdh* gene were amplified from DNA using primers dGDH2_f1/dGDH2_r1 and dGDH2_f2/dGDHr (Supplementary Table S6) and cloned in the pCM184 vector at the BglII/Acc65I and SacII/SacI sites. The resulting vector pCM184:gdhKm was introduced into *Mtm. alcaliphilum* cells by electroporation^36^. The *gdh*^*─*^ clones were selected by PCR using primers dGDH2_f1 and dGDHr.

The *gntk-gnl* DNA locus was amplified by PCR using primers dGnKinF and dGnKinR (Supplementary Table S6) containing the recognition sites for restriction endonucleases EcoRI and MluI and inserted into pCM184 with removal of the kanamycin resistance gene. The middle of this locus was removed at the sites of endonuclease PvuI and replaced by the kanamycin resistance gene. The kanamycin resistance gene was amplified by PCR from pCM184. The resulting plasmid pCM184:gntk-Km was introduced into *Mtm. alcaliphilum* cells by electroporation.

To obtain double *gdh*^*─*^*/glk*^*─*^ or *gntk*^*─*^*/glk*^*─*^ mutant strains, the previously generated plasmid pCM184:glkGm^8^ was introduced into either *gdh*^*─*^or *gntk*^*─*^ strain by electroporation. The *glk*^*─*^ clones were selected by PCR using primers dGlk (Acc65I) and dGlk (SacII) (Supplementary Table S6). The *gdh*^*─*^*/glk*^*─*^ and *gntk*^*─*^*/glk*^*─*^ strains were selected on methanol plates with Km (100 µg/mL) and Gm (10 µg/mL).

### Isolation and analysis of carbohydrates

Carbohydrates were analyzed in cells of *Mtm. alcaliphilum* grown up to the late exponential phase in 750 ml flasks containing 200 mL of mineral salt medium at stirring. Cells of the WT strain grown under methane were collected at the optical density OD_600_ = 0.84–0.92 (which was equivalent 53–57 mg DCW), cells of *gntk*^─^/glk^─^ mutant at OD_600_ = 0.6–0.7 (42–45 mg DCW) and for *gdh*^─^/*glk*^─^ mutant at OD_600_ = 0.61–0.68 (40–43 mg DCW). The WT strain grown on methanol was harvested up to OD_600_ = 0.91–0.93 (which was equivalent to 57–60 mg DCW), cells of the *gntk*^─^/glk^─^ mutant to OD_600_ = 0.66–0.78 (43–47 mg DCW) and *gdh*^─^/*glk*^─^ strain to OD_600_ = 0.66–0.68 (41–43 mg DCW). The freeze-dried cells (30–50 mg) were used for carbohydrate extraction by 80% methanol as described^8^. The dried methanol extract was dissolved in 540 µL H_2_O followed by the addition of 60 µL 4 M solution of 3-trimethylsilyl [2,2,3,3-2H4] propionate (TSP) in D_2_O mixing in a shaker for 30 s and centrifuged at 15,000 g for 5 min. The spectra were recorded with an AVANCE III 600 NMR spectrometer (Bruker) at an operating frequency of 600 MHz, at 298 K, with a spectral width of 24 ppm and 90-degree pulse of 12.5 μs. The number of acquisitions was 128 scans; the interval between the scans was 10 s; free induction decay (FID) was recorded for 96 k points over 3.42 s. For correct signal assignment, both spectra acquired in the 1D and 2D COSY NMR experiments were analyzed. The data were confirmed using the AMIX spectral software database (Bruker), as well as the Biological Magnetic Resonance Data Bank internet-based collection.

The concentration of glucose 1-phosphate (δ 5.46, m, 1H), glucose 6-phosphate (δ 5.37, m, 1H), trehalose (δ 5.20, d, J = 3.8 Hz, 1H, 1′H), maltose derivative/maltotriose (δ 5.41, br 1,1′CH), glucose (δ 5.24, d, J = 3.8 Hz, 1H), sucrose (δ 5.42, d, J = 3.8 Hz, 1H) were measured by using H^1^NMR.The sucrose and glucose contents were additionally measured using anthron reagent^8^ and ABTS assay^37^. Analytical and NMR data for sucrose and glucose are practically coincided; Table 3 shows the data obtained by analytical methods.

The concentration of gluconate in methanol extract was determined using GntK from *Mb. luteus* (the present work). In brief, the reaction mixture (1 mL) contained 50 mM Tris–HCl buffer (pH 9.0), 10 mM MgCl_2_, 2 mM ATP, 0.3 mM NADP^+^, 5U 6PGDH, 10 U His-tagged GntK from *Mb. luteus* and 5–25 µL methanol extract. After 3-min incubation, OD_340_ was measured. To build the calibration curve, gluconate concentrations in the range of 2.925–23.4 µg/mL were used.

Glycogen was extracted from 20 mg of freeze-dried cells as described^31^. The glycogen content was estimated using “Glycogen Assay kit” according to the manufacturer's recommendations.

### Analysis of the promoter activity of the *gntk* and *amy* genes

Using primer pairs PamyF/PamyR or PglnF/PglnR (Supplementary Table S6) the PCR products of 563 and 551 bp containing the sequences of putative *amy* or *gntk* promoters, respectively, were obtained. The PCR product with the *cat* gene was generated with primers SDglnK + CmF/CmR using plasmid p34S-Cm as the source of the *cat* gene. Primers PamyF/PglnF and CmR are tailed with XbaI and SphI restriction ends, respectively. PamyR/PglnR is tailed with a 20 bp sequence homologous to the first 20 bp of SDglnK + CmF containing the SD sequence of the *gntK* gene. PCR products containing the *amy* or the *gntk* promoter and the *cat* gene were used as a template in the third PCR reaction to produce the final fusion PCR products P*amy*-*cat* or P*gntk-cat* with XbaI and SphI restriction ends. The plasmid pMHA200 (Supplementary Table S5) was digested with these endonucleases to remove a 752-bp segment, which was replaced by the P*amy*-*cat* or P*gntk-cat* fragments of DNA. The correct assembly was verified by sequencing, and the new plasmids were designated as pMHA200:P*amy*-*cat* and pMHA200:P*gntk-cat*. The resulting plasmids were introduced into *Mtm. alcaliphilum* cells by electroporation.

The activity of chloramphenicol acetyltransferase (i.e. catalyzis the reaction of the acyl group transfer from acetyl-CoA to chloramphenicol) was assessed by recording the 5-thio-2-benzoic acid accumulation as a result of interaction between 5,5′-dithiobis-(2-nitrobenzoic acid) (DTNB) and CoASH sulfhydryl groups at 30 °C. The reaction mixture (1 mL) contained 50 mM Tris–HCl buffer (pH 7.5), 0.1 mM DTNB, 1 mM acetyl CoA, and 10 − 50 μL cell-free extract of the *Mtm. alcaliphilum*. The reaction was triggered by 2 mM chloramphenicol. The optical density was measured at 412 nm. The *Mtm. alcaliphilum* pMHA:cat and WT strains lacked the activity of chloramphenicol acetyltransferase.

## Supplementary Information

Supplementary Information
