# Supplementary material for: Enzymes of an alternative pathway of glucose metabolism in obligate methanotrophs
Source: Sci Rep. 2021 Apr 22;11:8795. doi: 10.1038/s41598-021-88202-x (PMC8062543; doi:10.1038/s41598-021-88202-x)
Supplement: Supplementary file 1 — Supplementary Information [file 41598_2021_88202_MOESM1_ESM.pdf]

Supplementary Information

Enzymes of an alternative pathway of glucose metabolism in obligate methanotrophs

Olga N. Rozova[1], Galina A. Ekimova[1], Nikolai V.Molochkov[2], Alexander S. Reshetnikov[1], Valentina N. Khmelenina[1]*, Ildar I. Mustakhimov[1]

[1]Federal Research Center "Pushchino Scientific Center for Biological Research of the Russian Academy of Sciences", G.K. Skryabin Institute of Biochemistry and Physiology of Microorganisms, Russian Academy of Sciences, 142290 Pushchino, Moscow Region, Russia

[2]Institute of Theoretical and Experimental Biophysics, Russian Academy of Sciences, Pushchino, Moscow Region 142290, Russia

* Corresponding author
E-mail: khmelenina@ibpm.pushchino.ru

Supplementary Table S1. The effects of K^+, NH^+_4, Na^+ cations (50 mM) and divalent metals (1mM) on the activity of GDHs from methanotrophs

Cations	Residual activity, %			
	MtmGDH	MbGDH	MtmGntK*	MbGntK*
Without cations	100 ± 1	100 ± 1	<0.01	<0.01
K^+	103 ± 1	131 ± 1	72 ± 1	99 ± 1
Na^+	79 ± 2	128 ± 1	100 ± 2	135 ± 1
NH_4^+	166 ± 5	136 ± 1	70 ± 1	117 ± 1
Mg^{2+}	95 ± 1	117 ± 1	100 ± 2	100 ± 2
Co^{2+}	92 ± 1	14 ± 1	26 ± 1	127 ± 2
Cu^{2+}	92 ± 2	59 ± 1	2 ± 0.6	5 ± 0.5
Mn^{2+}	82 ± 1	54 ± 1	102 ± 2	135 ± 1
Ni^{2+}	87 ± 1	10 ± 0.2	10 ± 1	66 ± 1
Cd^{2+}	89 ± 1	3 ± 0.5	4 ± 0.6	18 ± 1
Zn^{2+}	62 ± 0.5	6 ± 0.2	2 ± 0.2	30 ± 1
Ba^{2+}(1)	n.d.	n.d.	2 ± 1	75 ± 1
Ca^{2+}(1)	n.d.	n.d.	43 ± 1	115 ± 1
Sn^{2+}(1)	n.d.	n.d.	82 ± 1	80 ± 1

*All reactions were carried out in the presence of 5 mM $MgCl_2$, except for "without cations".
n.d. – not determined

Supplementary Table S2. The effects of various metabolites on the activity of GDHs from methanotrophs

Effector (Concentration)	Relative activity (%)	
	Mtm. alcaliphilum	*Mb. luteus*
Without effector	100± 2	100± 1
Glucose-6-phosphate: 2.5 mM	102 ± 1	81 ± 1
5 mM		67 ± 1
10 mM		60 ± 1
Glucose-1-phosphate(5 mM)	103 ± 1	103 ± 1
Fructose-6-phosphate:2.5mM		93 ± 1
5 mM	101 ± 1	85 ± 1
10 mM		81 ± 1
Fructose-1,6-bisphosphate (5 mM)	103± 1	94 ± 1
Phosphoenolpyruvate (1 mM)	94 ± 1	99 ± 2
Pyruvate (1 mM)	96 ± 1	104 ± 1
Oxaloacetate (1 mM)	107 ± 3	108 ± 1
Isocitrate (1 mM)	140 ± 4	105 ±1
Citrate (1 mM)	119 ± 1	111 ± 3
Malate (1mM)	95 ± 5	113 ± 2
α-Ketoglutarate (1 mM)	104 ± 1	108 ± 1
Succinate (1 mM)	99 ± 1	115 ± 3
Lactate (1 mM)	116 ± 1	100 ± 1
Serine (1 mM)	108 ± 3	110 ± 2
ATP (1 mM)	93± 2	112 ± 1
ADP (1mM)	87 ± 1	110 ± 1
AMP (1mM)	109 ± 1	108 ± 2
PPi(1 mM)	100 ± 2	109 ± 1

Supplementary Table S3. The effects of various metabolites on the activity of methanotrophic GntKs

Effector (Concentration)	Relative activity (%)	
	Mtm. alcaliphilum 20Z	*Mb. luteus*
Without effector	100 ± 1	100 ± 1
Pyruvate (3)	95 ± 2	105 ± 1
ADP (5)	71 ± 1	97 ± 1
AMP (4)	98 ± 1	111 ± 2
6-Phosphogluconate (5)	81 ± 1	84 ± 2
Phosphoenolpyruvate (5)	84 ± 1	85 ± 1
α-Ketoglutarate (1)	90 ± 2	113 ± 1
Oxaloacetate (1)	101 ± 1	111 ± 1
Glucose 1-Phosphate (5)	96 ± 2	110 ± 1
Glucose 6-Phosphate (5)	96 ± 1	101 ± 2
PPi (2)	100 ± 1	105 ± 1

Supplementary Table S4. Distribution of some genes of carbohydrate metabolism in methanotrophs

**Methanotrophs**	***gdh/ gntK***	***gntD***	***glk (ppgk)***	***PTS***	***sps (or sus)***	***treT (treS)/ treA (treP)***	***treY/ treZ***	***glgA***
**Gammaproteobacteria**								
Methylobacter luteus **IMV-B-3098T**	+/+	+	+	-	+	-/-	-/+	+
Methylobacter marinus A45	+/+	-	+	-	+	*treT/treA*	+/+	+
Methylobacter sp. BBA5.1	+/+	-	+	-	+	*treT/treA*	+/+	+
Methylobacter tundripaludum SV96	+/-	+	++	-	+	-	+/+	+
Methylobacter whittenburyi ACM 3310	+/+	+	+	-	+	*treT/treA*	+/+	+
Methylocaldum szegediense O-12	+/+	-	+	-	+	*treT/treP*	+/+	++
Methylocaldum marinum S8	+/+	-	-	-	+	*treT/treA*	+/+	++
Methylomagnum ishizawai 175	+/+	-	+	-	+	-/-	-/-	+++
Methylogaea oryzae JCM 16910	+/-	-	+	-	-	-/-	-/-	+++
Methylococcus capsulatus Bath	-/-	-	+	-	-	-/-	-/-	++
Methylococcus capsulatus Texas	-/-	-	+	-	-	-/-	-/-	++
Methyloglobulus morosus KoM1	+/-	-	+	-	+	-/-	-/-	++
Methylohalobius crimeensis 10Ki	+/-	+	+	-	++	*treS/treA*	+/+ *ostB*	++
Methylomarinum vadi IT-4	+/-	-	+(+)	-	+	-/-	-/-	+
Methylomicrobium agile ATCC 35068	+/-	+	+	-	+	*treT*/-	+/+	+
Methylomicrobium album BG8	+/-	+	+	-	+	*treT*/-	+/+	+
***Methylotuvimicrobium alcaliphilum* 20Z**	+/+	-	+	-	+	*treS*/-	+/+	++
Methylotuvimicrobium buryatense 5G	+/+	-	+	-	+	-/-	-/+	++
Methylotuvimicrobium kenyense AMO1	- (fragment 43 a.a.)/-	-	+	-	+	*treS*/-	+/+	++
Methylomonas denitrificans FJG1	+/-	-	+(+)	-	+	-/-	-/-	++

Methylomonas methanica MC09	-/-	-	+(+)	-	+	-/-	-/+	+
Methylomonas methanica NCIMB 11130	+/-	-	+(+)	-	+	-/-	-/-	+
Methylomonas sp. 11b	+/-	-	+(+)	-	-	-/-	-/-	+
Methylomonas sp. MK1	-/-	-	+(+)	-	-	-/-	-/-	+
Methylomonas sp. LW13	+/-	-	+(+)	-	+	-/-	-/-	+
Methylosarcina fibrata AML-C10	+/-	+	+	-	++	*treT*** /*treA*	+/+	+
Methylomicrobium lacus LW14	+/-	+	+	-	-	*treT*/-	+/+	+
Methylovulum miyakonense HT12	+/-	-	+	-	-	-/-	-/-	+
**Alphaproteobacteria**								
Methylocapsa acidiphila B2	+/-	-	+	+	-	-/-	-/-	-
Methylocapsa aurea KYG	+/-	-	+	+	-	*treS*/*treA*	*otsAB*	+
Methylocella silvestris BL2	+/+*	+	+	+	-	*treS*/-	+/- *otsAB*	+
Methylocystis sp. SB2	-/-	-	-	+	-	-/-	-/-	+
Methylocystis parvus OBBP	-/-	-	-	+	-	-/-	-/-	+
Methylocystis rosea SV97T	+/-	-	-	+	-	-/-	-/-	+
Methylocystis sp. ATCC 49242	+/-	-	-	+	-	-/-	*otsAB*	+
Methylocystis sp. LW5	-/-	-	-	+	-	-/-	*otsAB*	-
Methylocystis sp. SC2	+/-	+	-	+	-	-/-	-/-	+
Methyloferula stellata AR4	+/-	-	+	+	-	*treS*/*treA*	+/+, *otsAB*	-
Methylosinus sp. LW3	-/-	-	-	+	-	-/*treP*	*otsAB*	-
Methylosinus sp. LW4	-/-	-	-	+	-	-/-	*otsAB*	-
Methylosinus sp. PW1	-/-	-	-	+	-	-/-	*otsAB*	-
Methylosinus trichosporium OB3b	-/-	-	-	+	-	-/-	*otsAB*	-
**Verrucomicrobia**								
Methylacidiphilum fumariolicum SolV	-/-	-	+	+	-	*treS*/+	+/+	+

Methylacidiphilum infernorum V4	-/-	-	+	+	-	*treS*/+	+/+	++
Methylacidiphilum kamchatkense Kam1	-/-	-	+	+	-	*treS*/+	+/+	+

The sequences from NCBI, IMG/MER and MicroScope databases (http://www.ncbi.nlm.nih.gov, https://mage.genoscope.cns.fr/, https://img.jgi.doe.gov/) were obtained by BLAST searches. *gdh*, glucose dehydrogenase; *gntK*, gluconate kinase; *gntD*, gluconate 2- dehydrogenase; *glk*, glucokinase; *ppgk*, polyphosphate-dependent glucokinase; *PTS*, enzyme of phosphotransferase system; *sps*, sucrose phosphate synthase; *sus*, sucrose synthase; *treT*, alpha,alpha-trehalose synthase; *treS*, maltose alpha-D-glucosyltransferase; *treA*, alpha,alpha-trehalase; *treP*, alpha,alpha-trehalose phosphorylase; *treY,* maltooligosyl trehalose synthase; *treZ,* trehalohydrolase; *otsA,* trehalose 6-phosphate synthase; *otsB,* trehalose 6-phosphate phosphatase; *glgA*, glycogen synthase. * gluconate transporter, ** trehalose transporter. Homologous enzymes are marked in red and blue.

Supplementary Table S5. List of plasmids used in this study.

Plasmid	Function and relevant characteristics	Reference
pHUE	Vector for Ubiquitin-His-tagged protein overproduction; Amp^R; T7lac	Catanzariti et al., 2004;
pET28	Vector for His-tagged protein overproduction; Km^R; T7lac	Novagen
pET28:gdhMtm	pET28 carrying *gdh* gene from *Mtm. alcaliphilum*	This work
pET28Ub-shHis	pET28 carrying ubiquitin-His-tagged gene	This work
pET28Ub-shHis:gdhMtm	pET28Ub-shHis carrying *gdh* gene from *Mtm. alcaliphilum*	This work
pET28Ub-shHis:gdhMb	pET28Ub-shHis carrying *gdh* gene from *Mb. luteus*	This work
pET30	Vector for His-tagged protein overproduction; Km^R; T7lac	Novagen
pET30:gntkMtm	pET30 carrying *gntK* gene from *Mtm. alcaliphilum*	This work
pET30:gntkMb	pET30 carrying *gntK* gene from *Mb. luteus*	This work
pCM184	ApR, KmR, TcR; broad-host range cre-lox allelic exchange vector	Marx , Lidstrom, 2002
pCM184:gdhKm	pCM184 carrying 3′- and 5′- fragments of *Mtm. alcaliphilum gdh* gene upstream and downstream the Km gene	This work
pCM184:gntk-Km	pCM184 carrying 3′- and 5′- fragments of *Mtm. alcaliphilum gntk* gene upstream and downstream the Km gene	This work
pCM184:glk-Gm	pCM184 carrying 3′- and 5′- fragments of *Mtm. alcaliphilum glk* gene upstream and downstream the Gm gene	Mustakhimov et al., 2017
p34S-Cm	Source of Cm-cassette	Dennis, Zylstra, 1998
pMHA200	IncP, ColE1; reporter GFP	Theisen et al., 2005
pMHA200:P*amy-cat*	pMHA200, with cat gene under control the putative promoter regions of *Mtm. alcaliphilum amy* gene	This work
pMHA200:P*gntk-cat*	pMHA200, with cat gene under control the putative promoter regions of *Mtm. alcaliphilum gntK* gene	This work

Supplementary Table S6. The primers used in the work. The restriction endonuclease sites are underlined

Primers	Target	Sequence (5’-3’) with indication of restriction endonuclease sites
GluDH-20Z-F (pET28)	Cloning of the *Mtm. alcaliphilum gdh* gene in pET28 His6-tag at the N-terminus	TTCATATGCCGCTTATTATCGAGAA (NdeI)
GluDH-20Z-R (pET28)		TAAGCTTACCCGCCTTCGCGAAAGC (HindIII)
Ub-F	Cloning of the gene encoding ubiquitin in pET28 – resultant vector - pET28Ub-shHis	TTCCATGGGCCATCATCATCATCATCACCATATGCAAATCTTTGTG (NcoI)
Ub-R		TTCCGCGGAGGCGCAACACCAGGT (SacII)
GDH-20Z-F(SacII)	Cloning of the *Mtm. alcaliphilum gdh* gene in pET28Ub-shHis - His6-tag and ubiquitin at the N-terminus	TCCGCGGTGGAATGCCGCTTATTATCGAG (SacII)
GDH(Mlut98)-F	Cloning of the *Mb. luteus gdh* gene in pET28Ub-shHis - His6-tag and ubiquitin at the N-terminus	TCCGCGGTGGAATGCCATTACTGATCGAAAAGAC (SacII)
GDH(Mlut98)-R		TGAATTCAGCCGCCCTCGCGAAATC (EcoRI)
MtmGntk-F	Cloning of *Mtm. alcaliphilum gntk* gene in pET30	TCATATGGATGGCGCGCAAGAAAATCGC (NdeI)
MtmGntk-R		TAAGCTTCGGCGAAAGACGGGTCGAGC (HindIII)
MbGntk-F	Cloning of *Mb. luteus gntk* gene in pET30	TCATATGAATAACGAATTTGATGAC (NdeI)
MbGntk-R		TAAGCTTCAGGCCTAGATAAACCATTAT (HindIII)
dGDH2_f1	Upper flanking region of the *gdh* gene in *Mtm. alcaliphilum*	ATAGATCTGTTGCACTGCGACGAAATG (BglII)
dGDH2_r1		AAGGTACCGGCATCGGACATATAATTGA (Acc65I)
dGDH2_f2	Lower flanking region of the *gdh* gene in *Mtm. alcaliphilum*	TACCGCGGAAGCCGATTTACTGAA (SacII)
dGDHr		TT*GAGCTC*TATCCGTTGGCTGCCGA (SacI)
dGnKinF	Cloning of the *gntk-gnl* DNA locus	ATGAATTCGGTCGATGAAACGGTCT (EcoRI)
dGnKinR		TTACGCGTTCTTTTCCGCATTCCAGT (MluI)
PamyF	Cloning a DNA locus containing a putative promoter region of the *amy* gene	ATTCTAGATCCGCTCGTTGTCCAAA (XbaI)
PamyR		ATTTGACTCGTCAGTACTGAGTTGTGTTTTCGT
PglnF	Cloning a DNA locus containing a putative promoter region of the *gntk* gene	TATCTAGACTGGCGCGTTTTCTCGA (XbaI)
PglnR		ATTTGACTCGTCAGTCCGGC
SDglnK+CmF	Cloning of the *cat* gene	ACTGACGAGTCAAATATGGAGAAAAAAATC
CmR		AA*GCATGC*ATTAATCAGGCGTAGCAACCAGGCGT (SphI)

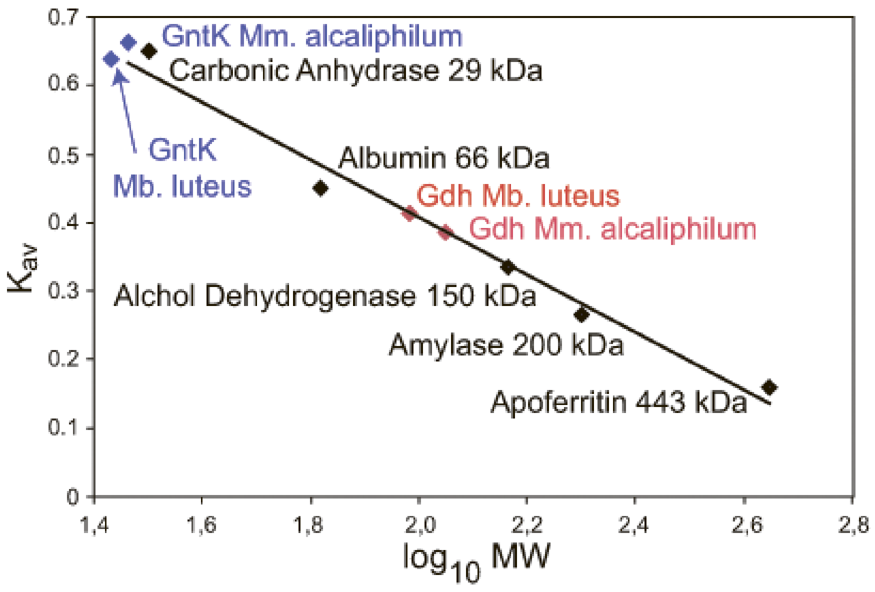

Supplementary Figure 1S. Oligomeric state determination of the recombinant enzymes from *Mtm. alcaliphilum* and *Mb. luteus* by using gel filtration chromatography. The proteins were detected by monitoring their absorbance at 280 nm.

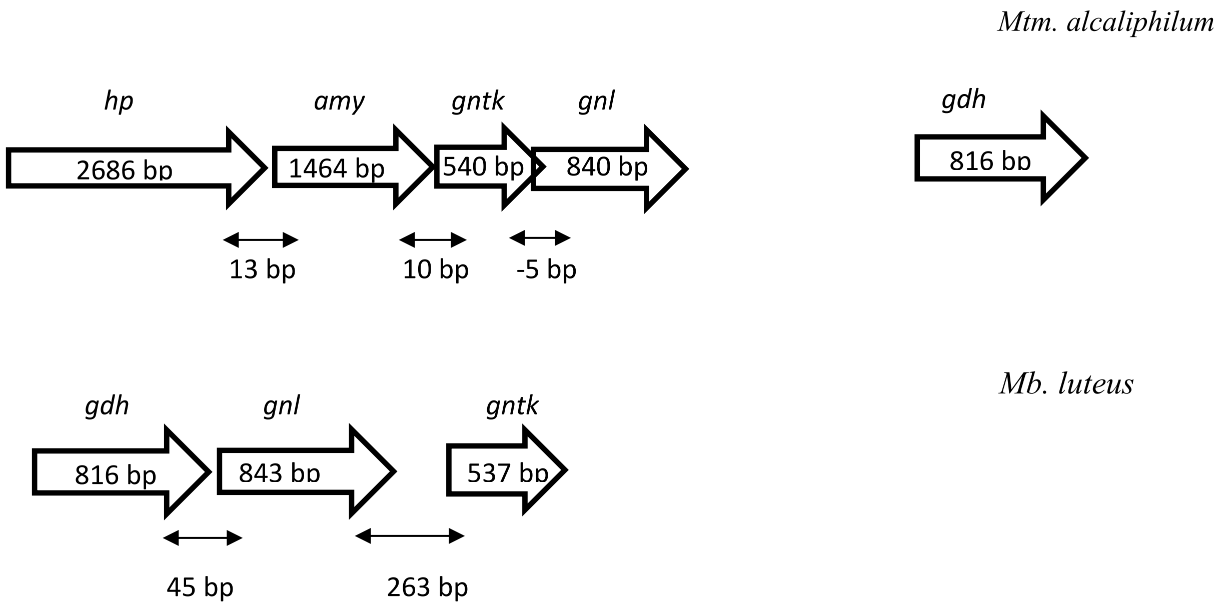

Supplementary Figure 2S. Location of the *gdh* and *gntk* genes in the chromosome of *Mtm. alcaliphilum* and *Mb. luteus*. *hp* – hypothetical protein, *amy* – D-Glucan glucanohydrolase (alpha amylase), *gntk* – gluconate kinase, *gnl* – gluconolactonase, *gdh* – glucose dehydrogenase.

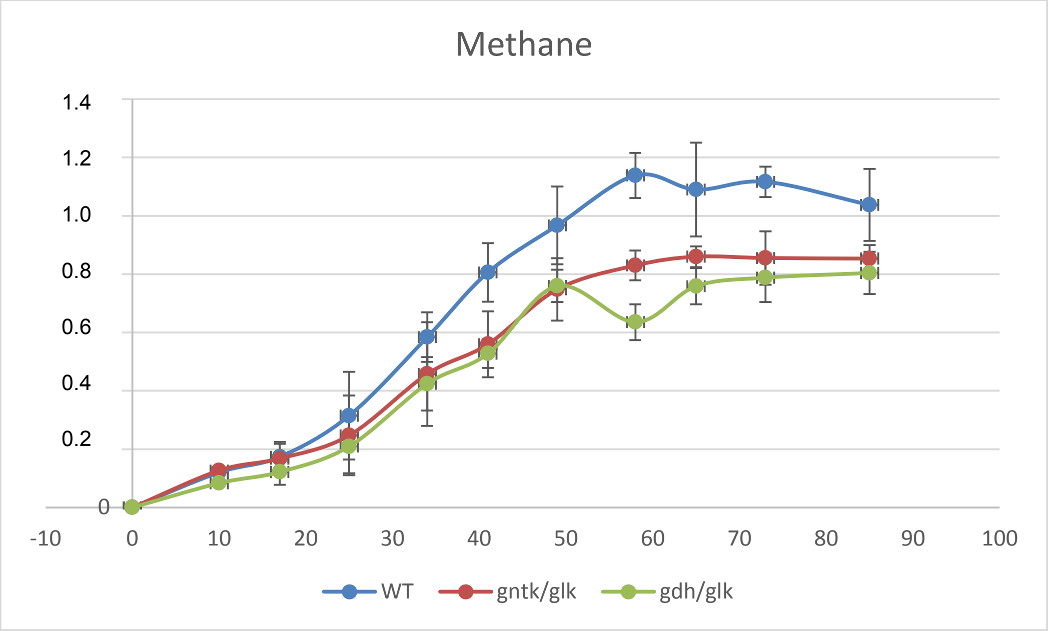

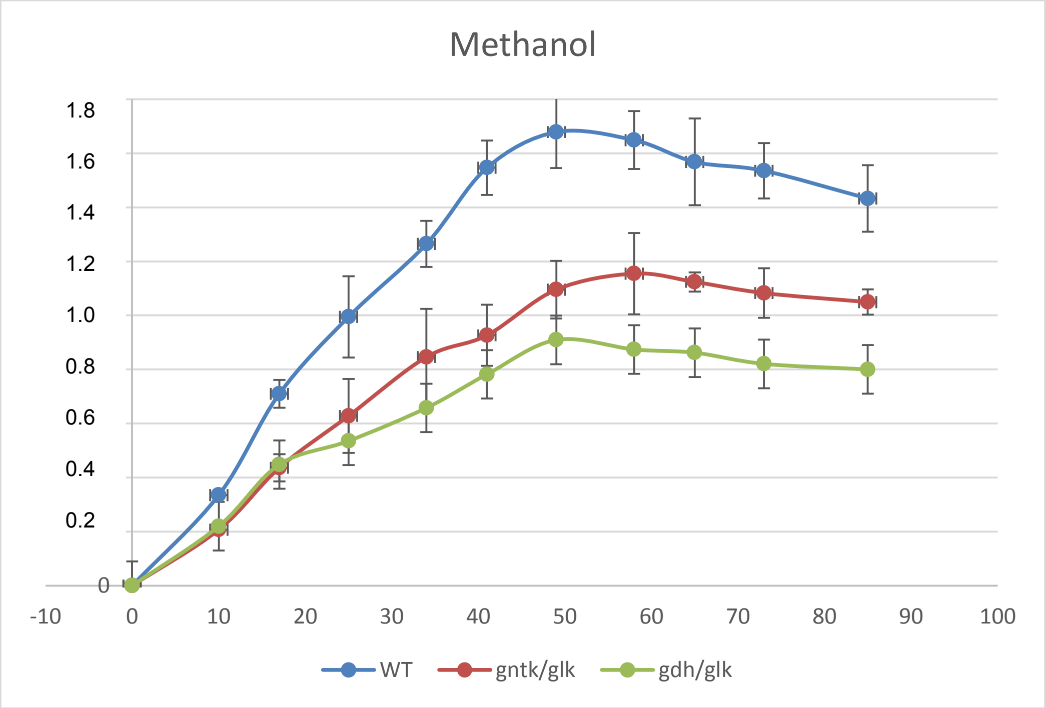

Supplementary Figure 3S. The growth of *Methylotuvimicrobium alcaliphilum* 20Z (blue line), *gdh⁻/glk⁻* (green line) and *gntk⁻/glk⁻* (red line) mutant cells in the presence of 3% NaCl.

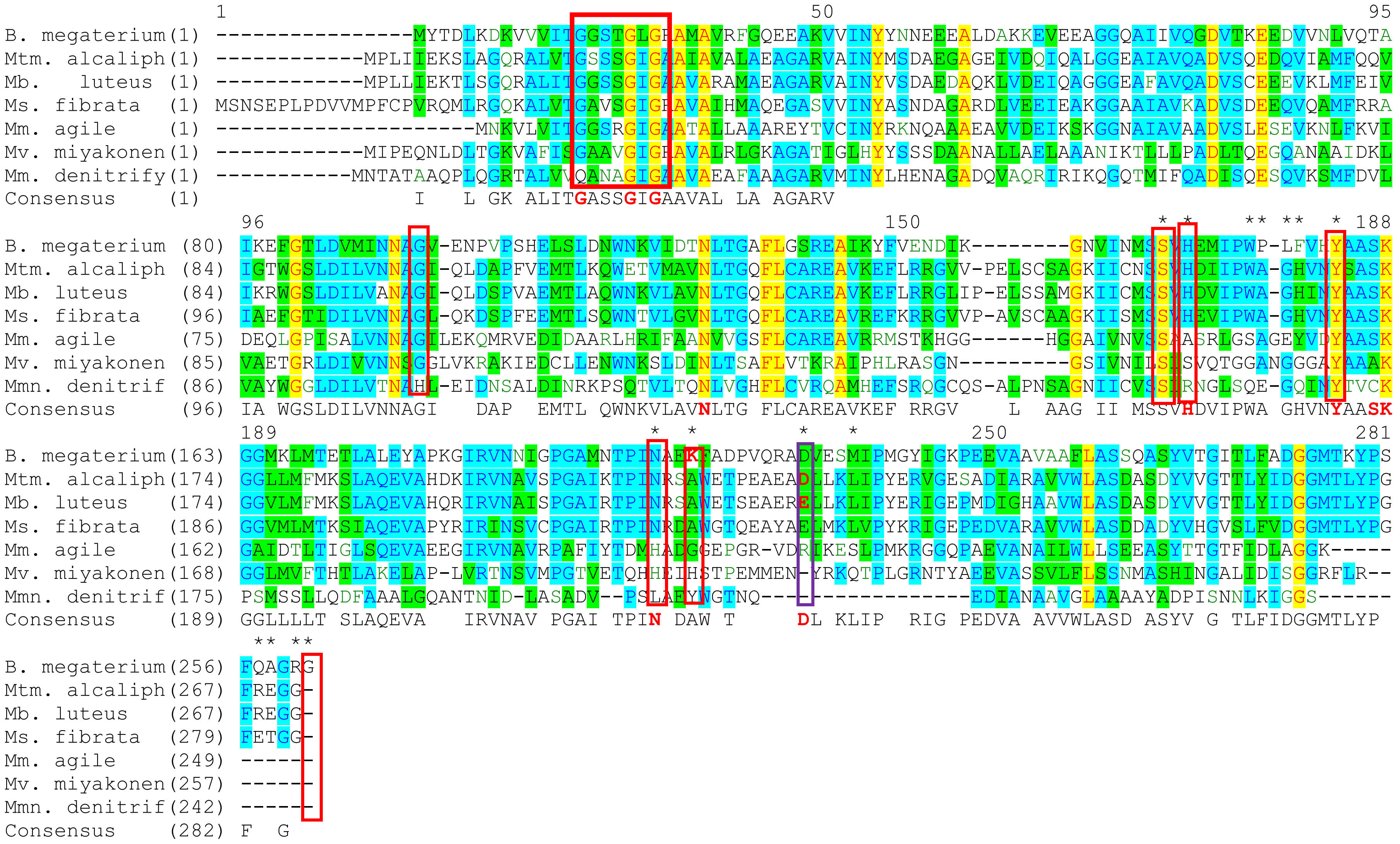

Supplementary Figure 4S. Multiple alignment of the primary structure of glucose 1-dehydrogenases. The square denotes Rossmann fold and amino acid residues forming hydrogen bonds with D-glucose. The asterisk indicates amino acid residues forming active site. Alignment is done using the Align tool by Vector NTI (Invitrogen Corparation)

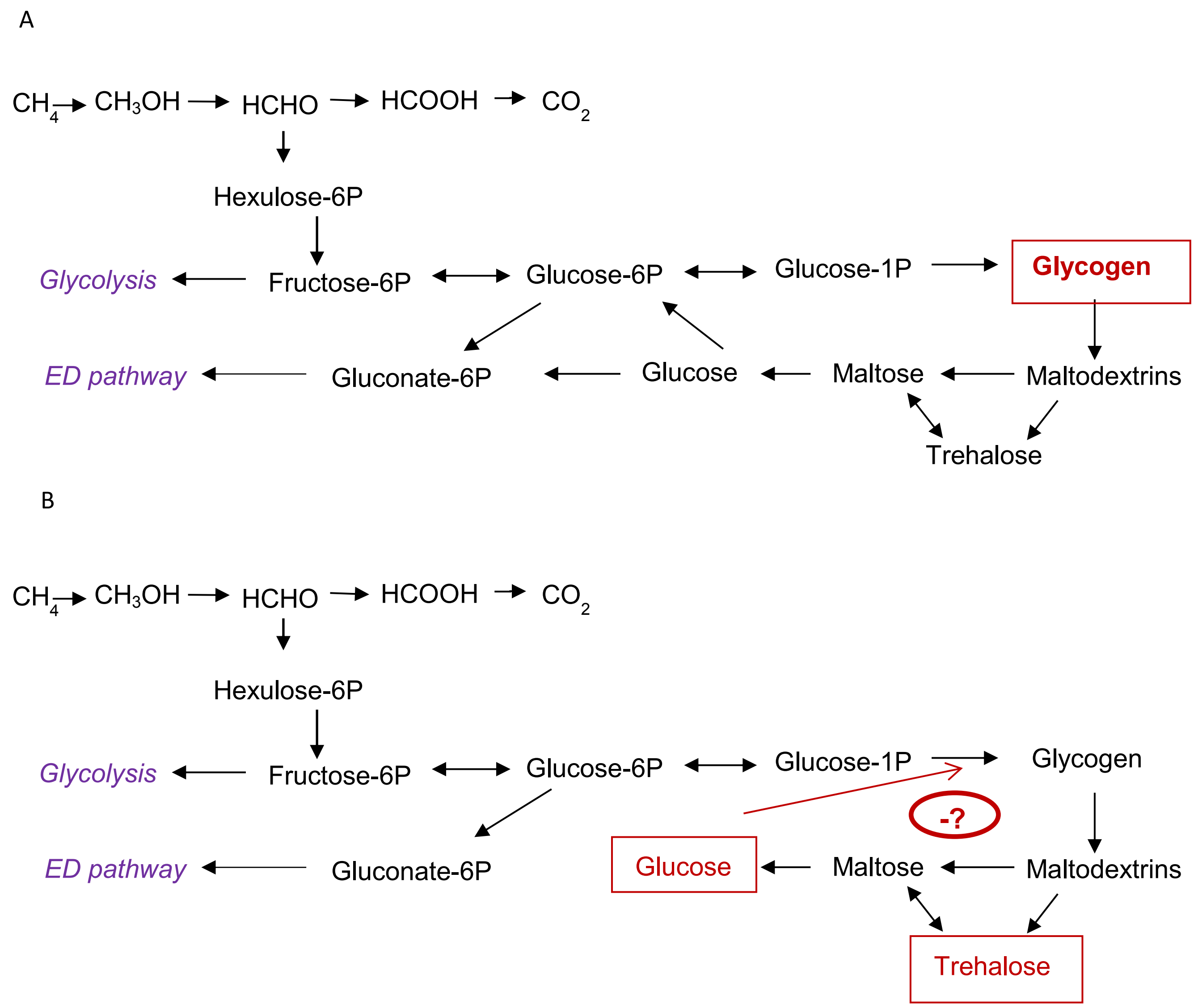

Supplementary Figure 5S. Schematic representation of the carbohydrate metabolism of *Mtm. alcaliphilum* 20Z (A) and *gdh*[−]/*glk*[−] mutant (B).

References

1. Catanzariti AM, Soboleva TA, Jans DA, Board PG, Baker RT. An efficient system for high-level expression and easy purification of authentic recombinant proteins. Protein Sci. 2004;13(5):1331-1339. doi:10.1110/ps.04618904.
2. Marx CJ, Lidstrom ME. Broad-host-range cre-lox system for antibiotic marker recycling in gram-negative bacteria. Bio Techniques. 2002; 33:1062–1067.
3. Dennis, J. J., and Zylstra, G. J. 1998. Plasposons: Modular self-cloning minitransposon derivatives for rapid genetic analysis of gram negative bacterial genomes. Appl. Environ. Microbiol. 64:2710-2715
4. Theisen, A.R.; Ali, M.H.; Radajewski, S.; Dumont, M.G.; Dunfield, P.F.; McDonald, I.R.; Dedysh, S.N.; Miguez, C.B.; Murrell, J.C. Regulation of methane oxidation in the facultative methanotroph Methylocella silvestris BL2. Mol. Microbiol. 2005, 58, 682–692.
